# Distribution, inequalities and associated factors of unmet medical needs among older adults: evidence from the national health service survey

**DOI:** 10.1186/s12877-026-07948-z

**Published:** 2026-07-09

**Authors:** Qingwen Deng, Yi Yang, Jingyi Qiao, Cunku Wang, Shuanghuai Cheng, Liya Fu, Yingyao Chen

**Affiliations:** 1 School of Public Health, Fudan University, No.138, Yi Xue Yuan Road, Shanghai, 200032 China; 2 National Health Commission Key Laboratory of Health Technology Assessment (Fudan University), Shanghai, China; 3 Statistical Information Center of Hainan Provincial Health Commission, Hainan, China; 4 Hainan Medical University Public Research Center, Hainan, China

**Keywords:** Unmet medical needs, Health inequalities, Decomposition analysis, Anderson model, Older adults

## Abstract

**Background:**

Analyzing and addressing the unmet medical needs (UMNs) of older adults is one of the key agendas for health systems to achieve the goals of universal health coverage. This study aims to investigate the distribution, determinants, and inequalities of UMNs among Chinese older adults.

**Methods:**

The data used from the 2023 National Health Service Survey in Hainan, with a sample of 4112 adults aged 60 and above. Chi-square tests and binary logistic regression were used to examined the factors associated with UMNs. Concentration indices and concentration curves to reflect the health inequalities in UMNs, and a decomposition approach was further employed to examine the contribution of each factor to these inequalities.

**Results:**

21.60% of the sample reported UMNs. Compared to the reference group, older adults who are of the Li ethnic group, had no personal income, did not participate in social activities, had no access to aging services, had a higher number of chronic diseases, and not physically independent had a higher likelihood of UMNs (*P* < 0.05). Additionally, socioeconomic-related health inequalities existed in the UMNs of the older adults, with a higher concentration among those with poorer economic condition, lower educational level, and those living in the western region (*P* < 0.01). The top three contributing factors to these inequalities were ethnicity (-47.03%), number of chronic diseases (-36.93%), and household per capita income (-25.36%).

**Conclusions:**

The UMNs of the older adults and the potential threat to inequalities require the attention of policymakers. Interventions should focus on improving access to medical services, with a particular emphasis on addressing the needs of vulnerable populations.

## Background

Achieving universal health coverage (UHC) is the goal and key agenda of health systems. The essence of UHC lies in people can access healthcare equitably their needs, regardless of their ability to pay or social background [1]. In practice, unnecessary, avoidable, and inequitable disparities in healthcare access caused by social determinants such as income, education, gender, and geographic location are a persistent challenge [2]. These disparities undermine UHC, as the failure to use healthcare services when needed can lead to worsening health conditions, catastrophic health expenditures, and an exacerbation of health inequalities [3, 4]. To effectively assess the functioning of healthcare systems in addressing these challenges, it is crucial to examine indicators like “unmet medical needs (UMNs)” [5]. UMNs are defined as the subjective or objective perception of not receiving appropriate medical care when needed, aiming to capture the inconsistency between need and supply, which may reflect the vulnerabilities of healthcare and social protection systems [3]. As an indicator, UMNs reveal the gap between the healthcare services that individuals should receive based on professional judgement or normative standards and those they actually receive, thereby providing a more accurate assessment of the accessibility and equity of healthcare systems than measures of healthcare utilization alone [6]. It should be noted that, given the scarcity of resources, some degree of UMNs may be inevitable. What warrants particular attention is whether UMNs are inequitably distributed or systematically associated with individuals’ socioeconomic characteristics, as such patterns would signal structural failures in the healthcare system rather than merely reflecting resource constraints [7].

Currently, global population aging is advancing rapidly. Older adults have more disabilities, physical illnesses, and complex needs compared to younger populations [8]. Beyond their objective medical needs, they may also face a higher risk of being unattended, financially unsupported, or immobile [9]. This inevitably leads to an increase in the number of older adults with UMNs, as current health and social systems continue to face challenges [10]. Previous studies have also pointed out that the prevalence of UMNs is higher among the older population [9]. To some extent, the relationship between socioeconomic inequality, health vulnerability, and UMNs is cyclical [3]. The accumulation of socioeconomic disadvantages and health disadvantages throughout the life course exacerbates UMNs in older adults, while UMNs are further associated with poverty, leading to inequitable access to medical services [11]. The UMNs of the older population therefore deserve attention, as meeting their needs can prevent health deterioration, which is essential for achieving healthy aging and universal health coverage.

Previous studies have proposed various explanations for UMNs, including deficiencies in the healthcare system, such as shortages of medical services or unavailable treatments, as well as personal circumstances of patients, such as affordability, mild symptoms, inconvenient transportation, or lack of time [7, 12–15]. For instance, Lee et al. [14] examined the impact of UMNs caused by “economic burden” and “lack of free time”; Allin et al. [7] explained UMNs from three aspects: waiting time, barriers, and personal reasons; Bryant et al. [12] emphasized that an important aspect of unmet need is waiting for care; Hansoo [16] categorized UMNs into four types: unmet due to economic reasons, unmet due to availability (lack of free time, long waiting lists, or lack of family doctors), unmet due to mild symptoms, and unmet due to other (not specified) reasons. In general, they can be conceptualized into several categories, including accessibility (financial or transportation barriers), availability (long waiting times, lack of services, etc.), acceptability (being busy or neglecting health issues [17]), and receiving services that did not meet expectations [3, 7].

A growing body of research has explored UMNs and their influencing factors. In terms of demographics, the likelihood of UMNs is higher in groups such as females, ethnic minorities, and those with a lower level of education, etc. For example, Lucevic et al. confirmed that the most vulnerable groups at risk of UMNs were those with only primary or secondary education and those from the lowest income quintile [18]. With regard to the factors to an individual’s ability to access health care, socio-economic conditions and the socio-economic environment in which they live make the greatest contribution. For instance, Yoon et al., using data from the Ministry of Health and Public Welfare of Korea, found that 11.6% of Korean adults experienced UMNs, with the most significant reason being financial barriers [5]. Lim et al. presented the results of a study conducted by the United Nations Development Programme and the World Health Organization, which examined outpatient service utilization in three provinces of China. They defined those who had not entered the healthcare system throughout the year, despite feeling the need to do so, as the UMNs group, accounting for 16% of the total population. Their analysis model showed that significant predictors for this subgroup were poorer provinces, lack of health insurance, and lower household income [19]. A study from Canada indicated that 12.8% of Canadians reported UMNs in the previous year [20]. Multiple studies have shown that low affordability, low acceptability, and perceived inadequacy of medical services are the main reasons for UMNs. For instance, American-based research found that the two strongest correlates of UMNs were lack of insurance and low income [21–23]. Li et al. reported that social health insurance could significantly reduce forgone care in both outpatient and inpatient settings [24]. Some studies also found that regional factors could determine individuals’ UMNs. Heo et al. found a negative correlation between the number of regional medical resources and UMNs [25]. Living on an island was also associated with UMNs [26]. Finally, an individual’s level of health also affects the probability of UMNs. Poorer health conditions can increased the likelihood of UMNs [27–30]. Momtaz et al. suggested that disabled older adults and those with chronic illnesses are more likely to report UMNs [31]. Mielck et al. found that older adults with poorer self-reported health and lower incomes are more likely to report UMNs [32].

China is undergoing rapid population aging, with the proportion of people aged 60 and above expected to rise to 28% by 2040 [3]. It has been reported that the proportion of older adults in China suffering from multiple diseases and functional impairments is continuously increasing [33], especially among socioeconomically disadvantaged groups. Despite the fact that China’s medical insurance system has largely achieved universal coverage and has played an important role in enhancing the healthcare security of the older population, UMNs may still persist. The primary function of medical insurance lies in alleviating patients’ financial burdens and promoting access to healthcare services. However, the depth and breadth of insurance coverage have not fully addressed all the challenges faced by the older adults. For instance, limitations in the scope and reimbursement rates of insurance, uneven distribution of medical resources between urban and rural areas, and constraints in health literacy and awareness continue to be potential barriers to accessing adequate healthcare services. As vulnerabilities increase, so does the need for medical services, and consequently, UMNs. The 2013 National Health Service Survey showed that as many as 27% of older adults were affected by UMNs [34]. Despite this concerning result, research on UMNs among older adults in China remains limited. Gao et al. analyzed and reported the reasons for UMNs among middle-aged and older adults, including financial difficulties, low acceptability, and insufficient healthcare resources [3]. However, few studies have analyzed inequalities in UMNs among older adults. Most previous research has focused on medical service utilization and its inequalities, pointing out a pro-rich inequality in the utilization of medical services among the older adults.

Overall, the current healthcare system is insufficient to meet the needs of the older adults [35]. Exploring UMNs and the associated inequalities can help understand the scale and nature of the medical service gaps in an aging society, which is crucial for achieving universal health coverage. To date, most studies on UMNs have focused on measuring its prevalence and investigating its predictors, with little attention given to inequalities in UMNs. There is limited evidence on how UMNs among Chinese older adults vary by individual socioeconomic status, health conditions, and environment [36]. This study aims to examine the prevalence and associated factors of unmet medical needs among older adults in China and to quantify socioeconomic-related inequalities in these needs through a decomposition approach.

## Methods

### Data source and sample

Data were obtained from the 2023 seventh wave of National Health Service Survey (NHSS) of Hainan, China. The National Bureau of Statistics conducts the NHSS every five years using a multi-stage stratified cluster sampling method. The survey covers 31 provinces, 156 counties, 780 towns, and 1,560 villages, with 60 households selected from each sampled village. A total of 93,600 households nationwide participated in the survey [2]. From Hainan Province, 156 counties (cities, districts) were randomly selected, and 60 households were randomly drawn from the village (community) committees in each sample county, resulting in 6,480 households in total. The survey subjects were the actual members of the sampled households. During the survey, interviewers conducted face-to-face interviews using mobile phones or tablets following the electronic questionnaire. The NHSS survey content included residents’ health status, healthcare service needs and utilization, levels of medical insurance, and perceptions of healthcare burden. To ensure the quality of the survey, supervisors guided the entire process, and the provincial, municipal, and district health commissions re-examined a certain number of survey participants. As the target population of this study is the older adults, participants under the age of 60 were excluded. A total of 4,112 samples were included in the final analysis.

Ethical approval for the National Health Service Survey was granted by the institutional review board of the Chinese National Bureau of Statistics. This study utilized de-identified secondary data provided by the Statistical Information Center of Hainan Provincial Health Commission; therefore, additional ethical approval was not applicable. Oral consent was obtained from all participants prior to the survey.

### Measures

The outcome variable is UMNs, measured by asking whether the respondent ever felt they needed outpatient or inpatient services due to illness or injury but did not use them in the past 12 months. The response was coded as a binary variable: 0 for “No” and 1 for “Yes.” The reasons for UMNs were also investigated. Respondents were asked to explain why they did not seek outpatient or inpatient services. The response options were as follows: (1) perceived the illness as mild/no need; (2) no effective treatment available; (3) financial difficulties; (4) poor service quality; (5) lack of time; (6) no available hospital beds; (7) restrictions of medical insurance; and (8) other reasons. Based on the conceptualization of UMNs and previous research [7, 16], the UMNs were categorized into four types: financial difficulty (unmet due to economic reasons), including options 3 and 7; access difficulty (unmet due to availability, uncontrollable barriers such as time and transportation, long waiting lists, or unavailable services), including options 2, 5, and 6; perceived dissatisfaction (general dissatisfaction with outpatient or inpatient experiences), including option 4; and other (unmet for other reasons, such as perceived mild illness), including option 1 and 8.

We adopted the Andersen Healthcare Utilization Model to select factors related to the prevalence of UMNs. The Andersen model is a widely used theoretical model in health research to study influencing factors, which include predisposing factors, enabling factors, and need factors [37, 38]. In this study, predisposing factors refer to demographic characteristics of the sample, including sex (male/female), age (60–69/70–79/80 and above), ethnicity (Han/Li/Other), marital status (married/other, including divorced, widowed, and never-married), and education level (illiterate/primary school/middle school/high school/college and above). Ethnicity was included as a unique predisposing factor given Hainan’s distinct ethnic composition. The Han ethnic group constitutes the majority population of China, while the Li ethnic group is the indigenous ethnic minority of Hainan Province, predominantly residing in the central and southern mountainous areas of the island and accounting for the largest non-Han population in the province. The Li population is characterized by distinct cultural traditions, language, and patterns of geographic settlement, which may shape health literacy, healthcare-seeking behaviors, and the cultural adaptability of medical services.

Enabling factors refer to the ability to access healthcare services, including region (eastern/central/western), medical insurance (yes/no), per capita household income (quartiles), distance to the nearest healthcare facility (< 1 km/1–2 km/2–3 km/>3 km), participation in social activities (yes/no), monthly personal income (yes/no), and availability of aging services (yes/no). Participation in social activities was measured by a multiple-choice question asking respondents which of the following activities they had engaged in: community security patrols, caring for other older adults, environmental sanitation, dispute mediation, companionship, professional volunteer services, household chores, farming, individual or private economic activities, or others. Respondents who selected any of the listed activities were coded as “yes”, while those who selected “none” were coded as “no”. Availability of aging services was assessed similarly, by asking respondents whether they had received any of the following services: health education, medical assistance, rehabilitation and nursing care, psychological support, daily-living care, cultural and recreational activities, rental of assistive devices for older adults, education for older adults, free health check-ups, traditional Chinese medicine–based wellness services, or others. Respondents who reported receiving any of these services were coded as “yes”, and those who reported “none” were coded as “no”.

Need factors refer to an individual’s health status, including the number of chronic diseases (none/1/2/3 or more) and activities of daily living (ADL) (physically independent/needs assistance). The number of chronic diseases was assessed by asking respondents whether they had been diagnosed with any chronic conditions and, if so, to list the specific diseases by name. Reported conditions primarily included common chronic diseases such as hypertension, diabetes, cardiovascular and cerebrovascular diseases, and respiratory diseases. Responses were then categorized as none/1/2/3 or more. ADL was assessed using a scale that evaluates older adults’ independence in six functions: feeding, dressing, bathing, mobility, toileting, and bowel control [39]. If the response for any of these dimensions was “no”, the individual was classified as “needs assistance”.

### Analyses

The Chi-square tests were used to examine the differences of presence of UMNs and the reasons of UMN according to demographics. Binary logistics regressions were applied to explore the associated factors of the presence of UMNs, given UMNs are a binary variable. Model 1 included predisposing factors (sex, age, ethnicity, marital status, and education level), Model 2 additionally incorporated enabling factors (region, medical insurance, per capita household income, distance to nearest medical facility, personal income, social activity participation, and access to aging services), and Model 3 further added need factors (number of chronic diseases and ADL). All analyses were performed using Stata 17.0 software.

The concentration index (CI) was used to reflect inequalities in UMNs in each category. In this study, the CI was calculated separately using three variables: education level, per capita household income, and region. These variables were selected because they represent distinct but complementary dimensions of socioeconomic status that are widely recognized as key determinants of health inequalities. Education level captures knowledge-related resources and health literacy, per capita household income reflects material living standards, and region reflects the broader socioeconomic environment and medical resource distribution. For the calculation of CI, individuals were ranked from lowest to highest by education level (from illiterate to college and above), by per capita household income (from Q1 to Q4), and by region according to the level of economic development (from western to central to eastern), respectively. CI can be calculated using the following formula [40]:

$$\:CI=\frac{2}{\mu\:\times\:cov(h,r)}$$

where *h* is the outcome of the health event (UMNs in this study), $\:\mu\:$ is the mean of * h*, and * r* is the fractional rank of individuals in the distribution used. The CI ranges between − 1 and + 1, and a value of zero denotes absolute fairness. If the CI takes a positive value, it means that UMNs are more concentrated in the socioeconomic advantaged. Conversely, if the CI is negative, UMNs are more concentrated in the socioeconomically disadvantaged. The concentration curve is used to visually assess health inequalities. When the curve coincides with the 45° line, it indicates no inequality in UMNs, reflecting complete equity. A deviation from the 45° line suggests the presence of inequality in UMNs, with the extent of inequality increasing as the distance grows. If the curve lies below the equity line, UMNs are concentrated among groups with higher socioeconomic status (pro-rich); if the curve is above the equity line, UMNs are concentrated among groups with lower socioeconomic status (pro-poor).

Decomposition analysis was further performed to determine the contribution of each factor to the inequalities [41]. The starting point of the decomposition assumes that health outcomes are a linear function, where the health outcome y is expressed as a linear function of a set of * k* determinants:

$$\:y=\alpha\:+{\sum\:}_{k}{\beta\:}_{k}{x}_{k}+\epsilon\:$$

Wagstaff et al. (2003) represented the concentration index of health as the sum of contributions from various factors, such as population characteristics and socioeconomic status, along with an unexplained residual component, i.e., a two-part decomposition.

$$\:CI\left(y\right)={\sum\:}_{k}\left(\frac{{\beta\:}_{k}{\stackrel{-}{x}}_{k}}{\mu\:}\right){C}_{k}+\frac{G{C}_{\epsilon\:}}{\mu\:}$$

where $\:CI\left(y\right)$ is the standard concentration index, $\:{\stackrel{-}{x}}_{k}$ is the mean of the explanatory variable, $\:\mu\:$ is the mean of * y*, $\:{C}_{k}$ is the concentration index of each determinant, and $\:G{C}_{\epsilon\:}$ is the generalized concentration index associated with the error term $\:\epsilon\:$. On the one hand, the explained or deterministic component represents the effect of each variable * k* on health outcomes. The absolute contribution of each variable is calculated by multiplying the elasticity coefficient of each factor (i.e., the percentage change in health outcomes when the factor changes by 1%) by $\:{C}_{k}$. On the other hand, the unexplained or stochastic component represents the proportion of each variable’s contribution. This is obtained by dividing each variable’s contribution by the concentration index $\:{C}_{k}$ and multiplying by 100.

## Results

### Sample characteristics

Table 1 shows the characteristics of the included sample (*n* = 4112). The mean age of the sample was 69.67 years. The proportions of Han and Li ethnic were 69.34% and 27.16%, respectively. Most respondents were married (77.82%), had medical insurance (95.79%), lived in eastern region (57.30%), had engaged in social activities (65.44%), had access to aging services (81.57%), had no chronic diseases (57.39%), and physically independent (98.31%).

**Table 1:** Description of variables

	Frequency/Mean	Percentage (%)/SD
Sex		
Male	2080	50.58
Female	2032	49.42
Age	69.67	8.09
Ethnics		
Han	2855	69.43
Li	1117	27.16
Other	140	3.40
Marital status		
Married	3200	77.82
Other	912	22.18
Education level		
Illiterate	725	17.63
Primary school	1299	31.59
Middle school	1218	29.62
High school	754	18.34
College and above	116	2.82
Per capita household income		
Q1	1553	37.77
Q2	977	23.76
Q3	809	19.67
Q4	773	18.80
Medical insurance		
Yes	3939	95.79
No	173	4.21
Region		
Eastern	2356	57.30
Central	882	21.45
Western	874	21.25
Distance to nearest medical institution		
< 1 km	1411	34.31
1–2 km	1003	24.39
2–3 km	753	18.31
> 3 km	945	22.98
Personal income		
Yes	3876	94.26
No	236	5.74
Engaged in social activities		
Yes	2638	65.44
No	1393	34.56
Access to aging services		
Yes	3288	81.57
No	743	18.43
Number of chronic diseases		
0	2360	57.39
1	1339	32.56
2	356	8.66
3 or more	57	1.39
ADL		
Needs assistance	68	1.69
Physically independent	3963	98.31

### Distribution of unmet medical needs

The distributional differences of UMNs across various sociodemographic characteristics are revealed in Table 2 (*N* = 4112). The results show that older individuals are more likely to report UMNs if they belong to ethnic minorities, have lower education levels, lower per capita household incomes, lack medical insurance, reside in western regions, live farther from medical facilities, have no personal income, do not participate in social activities, lack access to aging services, suffer from multiple chronic diseases, and are not independent in activities of daily living (*P* < 0.05). Further analysis of the reasons for UMNs (*N* = 888) shows that 21.60% of the sample experienced UMNs. Whether UMNs were due to financial difficulties, availability, perceived dissatisfaction, or other reasons was mainly associated with education level, per capita household income, medical insurance, region, distance to nearest medical facilities, and number of chronic diseases (*P* < 0.05).

**Table 2:** Distribution and variations in UMNs

	Presence of UMNs (*N* = 4112)			Reason for UMNs (*N* = 888)				
	Yes	No	χ^2^	Financial difficulty	Access difficulty	Perceived dissatisfaction	Other	χ^2^
Gender			1.505					3.707
Male	433 (20.82)	1647(79.18)		210(48.5)	23(5.31)	87(20.09)	113(26.1)	
Female	455 (22.39)	1577(77.61)		242(53.19)	21(4.62)	71(15.6)	121(26.59)	
Age			1.229					5.766
60–69	515 (21.39)	1893(78.61)		275(53.4)	26(5.05)	85(16.5)	129(25.05)	
70–79	260 (22.61)	890(77.39)		127(48.85)	10(3.85)	52(20)	71(27.31)	
≥ 80	113 (20.4)	441(79.6)		50(44.25)	8(7.08)	21(18.58)	34(30.09)	
Ethnics			54.495***					8.768
Han	527 (18.46)	2328(81.54)		258(48.96)	25(4.74)	107(20.3)	137(26)	
Li	323 (28.92)	794(71.08)		172(53.25)	18(5.57)	43(13.31)	90(27.86)	
Other	38 (27.14)	102(72.86)		22(57.89)	1(2.63)	8(21.05)	7(18.42)	
Marital status			1.417					6.653
Married	210 (23.03)	702(76.97)		115(54.76)	12(5.71)	25(11.9)	58(27.62)	
Other	678 (21.19)	2522(78.81)		337(49.71)	32(4.72)	133(19.62)	176(25.96)	
Education level			17.154**					35.964***
Illiterate	195 (26.9)	530(73.1)		111(56.92)	10(5.13)	17(8.72)	57(29.23)	
Primary school	275 (21.17)	1024(78.83)		148(53.82)	11(4)	41(14.91)	75(27.27)	
Middle school	253 (20.77)	965(79.23)		125(49.41)	15(5.93)	58(22.92)	55(21.74)	
High school	147 (19.5)	607(80.5)		64(43.54)	6(4.08)	34(23.13)	43(29.25)	
College and above	18 (15.52)	98(84.48)		4(22.22)	2(11.11)	8(44.44)	4(22.22)	
Per capita household income			24.903***					47.253***
Q1	397 (25.56)	1156(74.44)		243(61.21)	14(3.53)	53(13.35)	87(21.91)	
Q2	197 (20.16)	780(79.84)		93(47.21)	10(5.08)	35(17.77)	59(29.95)	
Q3	143 (17.68)	666(82.32)		70(48.95)	8(5.59)	26(18.18)	39(27.27)	
Q4	151 (19.53)	622(80.47)		46(30.46)	12(7.95)	44(29.14)	49(32.45)	
Medical insurance			0.402					10.473*
Yes	854 (21.68)	3085(78.32)		429(50.23)	44(5.15)	158(18.5)	223(26.11)	
No	34 (19.65)	139(80.35)		23(67.65)	0(0)	0(0)	11(32.35)	
Region			10.824**					37.734***
Eastern	479 (20.33)	1877(79.67)		219(45.72)	24(5.01)	115(24.01)	121(25.26)	
Central	185 (20.98)	697(79.02)		109(58.92)	12(6.49)	25(13.51)	39(21.08)	
Western	224 (25.63)	650(74.37)		124(55.36)	8(3.57)	18(8.04)	74(33.04)	
Distance to nearest medical institution			9.834*					30.428***
< 1 km	324 (22.96)	1087(77.04)		140(43.21)	12(3.7)	66(20.37)	106(32.72)	
1–2 km	182 (18.15)	821(81.85)		85(46.7)	11(6.04)	41(22.53)	45(24.73)	
2–3 km	164 (21.78)	589(78.22)		93(56.71)	8(4.88)	28(17.07)	35(21.34)	
> 3 km	218 (23.07)	727(76.93)		134(61.47)	13(5.96)	23(10.55)	48(22.02)	
Personal income			19.595***					2.859
Yes	515 (19.52)	2123(80.48)		272(52.82)	27(5.24)	86(16.7)	130(25.24)	
No	356 (25.56)	1037(74.44)		172(48.31)	15(4.21)	70(19.66)	99(27.81)	
Engaged in social activities			7.888**					6.060
Yes	682 (20.74)	2606(79.26)		360(52.79)	32(4.69)	112(16.42)	178(26.1)	
No	189 (25.44)	554(74.56)		84(44.44)	10(5.29)	44(23.28)	51(26.98)	
Access to aging services			8.636**					3.717
Yes	819 (21.13)	3057(78.87)		411(50.18)	40(4.88)	151(18.44)	217(26.5)	
No	69 (29.24)	167(70.76)		41(59.42)	4(5.8)	7(10.14)	17(24.64)	
Number of chronic diseases			103.054***					31.171***
0	396 (16.78)	1964(83.22)		237(59.85)	15(3.79)	60(15.15)	84(21.21)	
1	352 (26.29)	987(73.71)		160(45.45)	21(5.97)	60(17.05)	111(31.53)	
2	109 (30.62)	247(69.38)		44(40.37)	7(6.42)	29(26.61)	29(26.61)	
3 or more	31 (54.39)	26(45.61)		11(35.48)	1(3.23)	9(29.03)	10(32.26)	
ADL			9.381**					1.450
Needs assistance	25 (36.76)	43(63.24)		14(56)	0(0)	4(16)	7(28)	
Physically independent	846 (21.35)	3117(78.65)		430(50.83)	42(4.96)	152(17.97)	222(26.24)	

***P < 0.001, ** *P* < 0.01, *P < 0.05

### Factors associated with UMN

Table 3 presents the results of binary regression analysis, incorporating various potential factors related to UMNs in sequential models. The final model (Model 3) indicates that, compared to the reference group, older individuals from the Li ethnic group (OR = 1.680, *P* < 0.001), those without personal income (OR = 1.248, *P* = 0.011), those not engaging in social activities (OR = 1.611, *P* = 0.009), those lacking access to aging services (OR = 1.340, *P* = 0.005), and those with a greater number of chronic diseases (OR = 1.728, *P* < 0.001; OR = 2.283, *P* < 0.001; OR = 6.374, *P* < 0.001) are more likely to experience UMNs. Compared to the reference group, older individuals aged 80 and above (OR = 0.748, *P* = 0.033), with a higher per capita household income (OR = 0.733, *P* = 0.003; OR = 0.643, *P* < 0.001; OR = 0.699, *P* = 0.003), those living 1–2 km from the nearest medical facility (OR = 0.753, *P* = 0.009), and those with independence in activities of daily living (OR = 0.511, *P* = 0.014) report a lower likelihood of UMNs.

**Table 3:** Binary logistic analysis of factors associated with UMN of the older adults (*N* = 4112)

	Model 1				Model 2				Model 3			
	OR	SE	*P*-value	95% CI	OR	SE	*P*-value	95% CI	OR	SE	*P*-value	95% CI
Gender (Ref: Male)												
Female	0.995	0.085	0.956	(0.843, 1.176)	1.031	0.091	0.729	(0.868,1.225)	1.043	0.093	0.641	(0.875, 1.242)
Age (Ref: 60–69)												
70–79	1.082	0.096	0.376	(0.909, 1.289)	1.081	0.099	0.397	(0.903,1.294)	1.021	0.095	0.822	(0.850, 1.227)
≥ 80	0.889	0.115	0.362	(0.689, 1.145)	0.849	0.112	0.215	(0.655,1.100)	0.748	0.102	0.033	(0.572, 0.977)
Ethnics (Ref: Han)												
Li	1.733	0.146	0.000	(1.469, 2.044)	1.771	0.157	0.000	(1.488,2.107)	1.680	0.152	0.000	(1.408, 2.006)
Other	1.475	0.299	0.055	(0.992, 2.194)	1.623	0.350	0.025	(1.064,2.476)	1.386	0.307	0.140	(0.898, 2.140)
Marital status (Ref: Married)												
Other	1.007	0.100	0.944	(0.830, 1.222)	1.056	0.106	0.592	(0.866,1.286)	1.067	0.109	0.524	(0.873, 1.304)
Education level (Ref: Illiterate)												
Primary school	0.747	0.086	0.011	(0.596, 0.936)	0.818	0.097	0.089	(0.649,1.031)	0.793	0.095	0.053	(0.627, 1.003)
Middle school	0.759	0.096	0.029	(0.592, 0.973)	0.857	0.114	0.247	(0.661,1.112)	0.842	0.113	0.200	(0.646, 1.096)
High school	0.736	0.106	0.033	(0.555, 0.975)	0.817	0.123	0.181	(0.608,1.098)	0.796	0.122	0.135	(0.590, 1.074)
College and above	0.602	0.169	0.070	(0.347, 1.043)	0.699	0.207	0.226	(0.391,1.248)	0.664	0.200	0.174	(0.369, 1.198)
Per capita household income (Ref: Q1)												
Q2					0.763	0.078	0.008	(0.625,0.931)	0.733	0.076	0.003	(0.598, 0.897)
Q3					0.680	0.077	0.001	(0.545,0.849)	0.643	0.074	0.000	(0.513, 0.805)
Q4					0.781	0.093	0.037	(0.619,0.985)	0.699	0.085	0.003	(0.551, 0.886)
Medical insurance (Ref: Yes)												
No					0.732	0.151	0.130	(0.490,1.096)	0.784	0.163	0.243	(0.522, 1.179)
Region (Ref: Eastern)												
Central					0.868	0.094	0.189	(0.702,1.072)	0.896	0.098	0.315	(0.722, 1.111)
Western					1.276	0.128	0.015	(1.048,1.553)	1.333	0.136	0.005	(1.092, 1.627)
Distance to nearest medical institution (Ref: <1 km)												
1–2 km					0.722	0.077	0.002	(0.585,0.890)	0.753	0.082	0.009	(0.609, 0.932)
2–3 km					0.847	0.096	0.141	(0.679,1.057)	0.871	0.100	0.229	(0.696, 1.091)
> 3 km					0.882	0.093	0.233	(0.718,1.084)	0.946	0.101	0.604	(0.768, 1.166)
Personal income (Ref: Yes)												
No					1.364	0.115	0.000	(1.157,1.609)	1.248	0.108	0.011	(1.053, 1.478)
Engaged in social activities (Ref: Yes)												
No					1.679	0.300	0.004	(1.183,2.383)	1.611	0.293	0.009	(1.127, 2.301)
Access to aging services (Ref: Yes)												
No					1.257	0.127	0.024	(1.031,1.533)	1.340	0.138	0.005	(1.095, 1.641)
Number of chronic diseases (Ref: 0)												
1									1.728	0.150	0.000	(1.458, 2.048)
2									2.283	0.306	0.000	(1.756, 2.969)
3 or more									6.374	1.790	0.000	(3.676, 11.053)
ADL (Ref: Needs assistance)												
Physically independent									0.511	0.140	0.014	(0.299, 0.873)

### Health inequalities in unmet medical needs

**Fig. 1:**
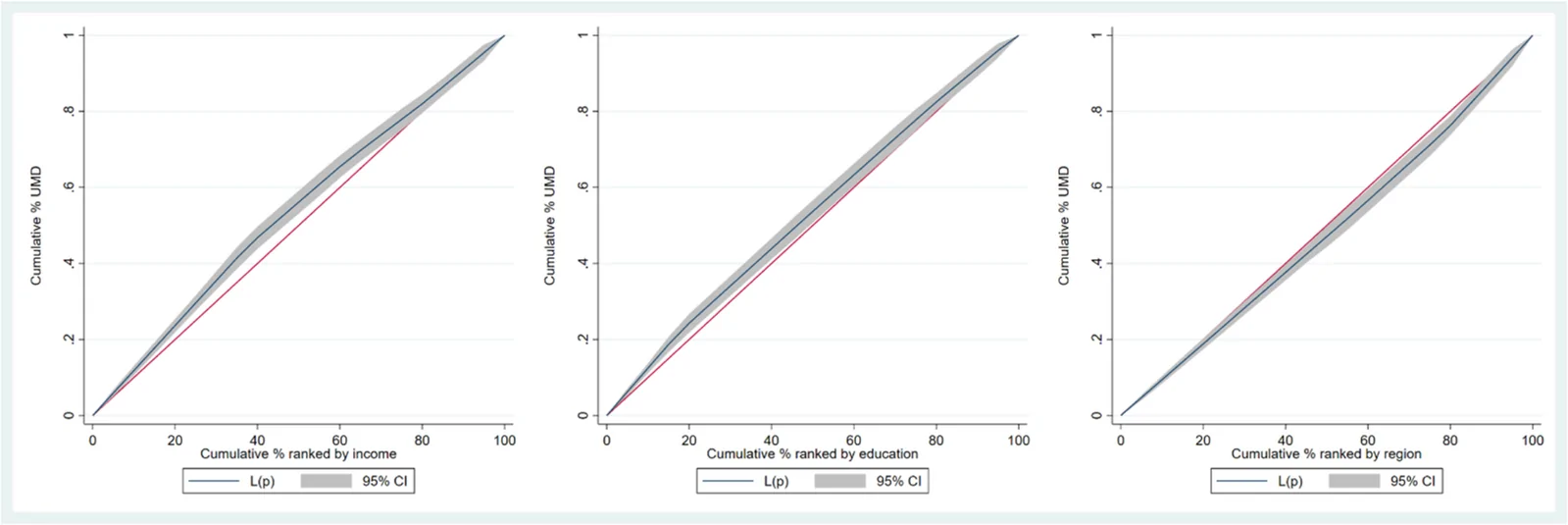
Concentration curves for socioeconomic-related inequalities in UMNs

Figure 1 illustrates the concentration curves for three potentially related variables of socioeconomic status: economic conditions (reflected by per capita household income), education level, and region, with concentration index (CI) values of -0.073 (*P* < 0.001), -0.058 (*P* < 0.001), and 0.043 (*P* = 0.005), respectively. The relative positions of the concentration curves and the equity line indicate the degree of inequality. The concentration curves for economic conditions and education level are both above their respective equity lines, indicating that UMNs are more concentrated among individuals with poor economic conditions and lower education levels. In contrast, the concentration curve for the regional variable is below the equity line, suggesting that UMNs are more concentrated among populations in the western regions. This indicates that there is inequality in the distribution of UMNs, which tends to affect those who are poorer, less educated, and living in underdeveloped areas.

Table 4 displays the results of the concentration index decomposition analysis. More specifically, the data provided records the relative contributions of each variable to the overall concentration index. We found that most of the observed pro-poor inequality in UMNs can be attributed to ethnicity, the number of chronic diseases, per capita household income, personal income, participation in social activities, independence in activities of daily living, and region, regardless of whether the inequality is income-related or education-related.

**Table 4:** Decomposition of the socioeconomic-related inequalities in UMN

Variable	Elasticity	Margin effects	CI	Contribution to income-related CI (%)	Contribution to education-related CI (%)
Gender	0.057	0.008	0.018	-1.416	-1.778
Age	-0.109	-0.014	0.002	0.298	0.375
Ethnics	0.343	0.054	0.100	-47.032	-59.089
Marital status	0.068	0.008	-0.015	1.392	1.748
Education level	-0.087	-0.007	-0.058	-6.938	-8.716
Per capita household income	-0.253	-0.023	-0.073	-25.362	-31.864
Medical insurance	-0.185	-0.037	-0.004	-1.017	-1.278
Region	0.163	0.021	0.043	-9.603	-12.065
Distance to nearest medical institution	-0.027	-0.002	0.001	0.037	0.046
Personal income	0.239	0.037	0.063	-20.674	-25.974
Engaged in social activities	0.414	0.082	0.033	-18.725	-23.526
Access to aging services	0.285	0.050	0.020	-7.823	-9.828
Number of chronic diseases	0.191	0.080	0.141	-36.929	-46.397
ADL	-0.946	-0.098	-0.012	-15.578	-19.572

## Discussion

The primary aim of this study is to analyze the determinants and inequalities associated with UMNs among the older adults in Hainan, particularly in the context of the country’s rapidly aging population, which is significant for achieving healthy aging and universal health coverage. While some scholars have examined healthcare utilization and inequalities among older adults, few have focused on the inequalities related to UMNs. Our analysis reveals that 21.60% of the sample experiences UMNs. The inequalities in UMNs primarily affects socioeconomically disadvantaged groups. Furthermore, the results of the decomposition analysis indicate that enabling factors (such as economic condition, education level, and social participation) and need factors (such as health status, including the number of chronic diseases and independence of activities of daily living) are the primary causes of these inequalities.

Our prevalence finding of 21.60% is higher than estimates reported in many previous studies, both within and outside China. Internationally, Yoon et al. reported that 11.6% of Korean adults experienced UMNs [5], Kasman and Badley found that 12.8% of Canadians reported UMNs in the previous year under a publicly funded health care system [15], and Lim et al. identified 16% of the general population as having UMNs in three Chinese provinces [14]. Wang et al. found that among middle-aged and older adults in Jiangsu Province in eastern China, 12.86%, 2.22%, and 48.89% reported UMNs for outpatient, inpatient services, and physical examinations, respectively [2]. Several reasons may explain these differences. First, differences in the study populations may be the primary reason for the variation in the prevalence of UMNs. The Korean, Canadian, and three-province Chinese estimates were derived from general adult populations, whereas our study is restricted to adults aged 60 and above. Compared to younger and middle-aged individuals, older adults are more sensitive to healthcare utilization and have greater demand for medical services due to their higher susceptibility to functional or cognitive impairments, higher prevalence of chronic diseases, and lower income. Previous studies have indicated that in contemporary China, the older subgroup is most likely to experience UMNs due to poverty, imbalanced distribution of medical resources, or lack of effective insurance [42]. Thus, studies focusing on older adults report higher prevalence rates than those focusing on other populations. Second, the comparator countries differ from China in the maturity and design of their healthcare and social protection systems. Korea and Canada both operate national health insurance schemes with relatively deep benefit packages and well-established long-term care arrangements for older adults, whereas China’s system, despite broad population coverage, still provides comparatively shallow reimbursement and limited integrated long-term care, which plausibly translates into a higher residual prevalence of UMNs; the fact that our study was conducted in Hainan, a tropical island province characterized by a dispersed population distribution across urban and rural areas, pronounced east to west disparities in the allocation of medical resources, and a large ethnic-minority population in less-developed inland areas, may further contribute to a higher observed prevalence. Third, the measures for UMNs vary. Previous studies based on the Chinese population typically measured UMNs by counting instances where “medical services were not used due to uncontrollable objective barriers to access” [2, 43], such as financial constraints, transportation difficulties, and time or service availability. However, the conceptualization of UMNs should also encompass patients’ distrust or dissatisfaction with medical services and their belief that clinical medicine cannot address their health problems, which reflects subjective unmet expectations or informed unchosen needs [3, 6]. This study takes this into account and adopts a broader definition of UMNs, resulting in a higher prevalence rate compared to other studies.

Regarding the factors related to UMNs and their inequalities, the predisposing factors include age and ethnicity. Compared to younger older-adults, the older older-adults (aged 80 and above) report a decrease in UMNs as they age. This is consistent with previous research findings [7] and may reflect changes in health-seeking behaviors and medical care utilization patterns throughout the life course [2], which can be understood from both objective and subjective perspectives. From an objective standpoint, although advanced age is typically associated with poorer health status and a stronger perceived need for medical care, the oldest old in China are also more likely to be retired or to have withdrawn from agricultural labour, and some may receive an old-age pension or family financial support, freeing them from the competing demands of employment or caregiving obligations, and to have greater discretionary time for seeking care; these life-stage circumstances reduce the practical barriers such as financial constraints, competing time demands, and caregiving obligations that typically generate forgone care among younger older adults, so that even when health needs are stronger, they are more likely to be acted upon and thus less likely to remain unmet [2]. From a subjective standpoint, as individuals approach the end of their life journey and physical functions decline, the older older-adults may reframe everyday symptoms and functional limitations as an inevitable component of senescence rather than as treatable disease, leading them to lower their internal threshold for what constitutes a “medical need” and to perceive fewer of their health problems as warranting professional care [7]. As described above, the Li ethnic group is the largest indigenous minority in Hainan, predominantly residing in the central and southern mountainous areas with relatively limited infrastructure and healthcare resources. In comparison to Han ethnicity, Li ethnicity is more likely to report UMNs. One reason for this may be that the Li ethnic group primarily resides in the central and southern parts of Hainan Province, where the more scarce medical resources in the region limit the fulfilment of medical needs, coupled with the persistently hot and humid tropical rainforest climate may pose potential health risks, leading to greater health needs. Differences in health literacy and healthcare-seeking behaviors between ethnic groups may also contribute to this disparity. The Li population’s health literacy may be shaped by distinct cultural perceptions of illness, traditional medical practices, and language-specific access to health information. For example, reliance on ethnomedical knowledge or limited exposure to mainstream health education materials might lead to divergent interpretations of symptoms and treatment options. Moreover, challenges such as insufficient cultural adaptation of medical services may reduce patient-provider communication effectiveness and service utilization [44, 45], causing patients to feel that their need are unmet.

Secondly, the enabling factors related to UMNs and their inequalities include region, economic condition, distance to the nearest medical institution, participation in social activities, and accessibility of aging services. The associations, particularly the higher likelihood of UMNs among older adults living in the western regions, those with lower per capita household income, those without personal income, those living 1–2 km from the nearest medical institution, those not participating in social activities, and those lacking access to aging services, can largely be attributed to financial constraints and medical service availability. The significant disparity in medical resources between regions is a major challenge currently faced by Hainan Province. In comparison to the eastern region, which accounts for more than half of the island’s population and possesses overwhelming amount of medical resources, the western region suffers from a severe shortage of medical resources, particularly tertiary hospitals capable of handling complex health issues, thus suppressing many potential health needs. Older adults who do not participate in social activities and lack access to aging services may experience greater feelings of loneliness and helplessness, triggering more medical needs. Previous research has confirmed a positive association between social participation and social support with cognitive function and mental health in older adults [46–48], as social participation and support can help mitigate negative emotions and enhance self-efficacy while maintaining physical and cognitive activity. They also provide a “buffer zone” to cushion the negative impacts associated with stressful events, thereby reaping potential health benefits.

This study presents an interesting finding regarding the impact of spatial accessibility to medical services on UMNs among the older adults. Specifically, compared to those who living less than 1 km from the nearest medical institution, those living 1–2 km away reported fewer UMNs, whereas those living more than 2 km away did not show significantly higher or lower levels of UMNs. There are possible explanations for this phenomenon. Although we might assume that the farther away from medical institution, the more UMNs there would be, this relationship may not be linear in practice. Older adults living within 1 km of medical institution may overuse healthcare services due to the very close proximity, making seemingly higher needs. The distance of 1–2 km, though slightly farther, remains within a relatively convenient range, where older individuals can still access medical services by walking or using appropriate public transportation. This distance may not be a significant barrier. Additionally, the slightly greater distance may prompt the older adults to plan their medical expectations and access more effectively, leading to fewer UMNs. For those living more than 2 km away from medical institution, the inconvenience of travel may indeed be a barrier; however, they may have adapted to these challenges over time and have developed other coping strategies, such as stockpiling commonly used medications, utilizing telemedicine services, or receiving regular assistance from family members. Furthermore, older adults living far from medical institutions may have lower expectations of healthcare and health awareness, so even if their needs are not fully met, their perception may not be as pronounced as those living closer to medical institutions. This finding may seem counterintuitive and warrants further investigation in future research.

Socioeconomic status, particularly economic income, has a significant impact on UMNs among the older adults. In other countries such as Greece, Italy, Poland, and Portugal, financial reasons are also the primary cause of UMNs [5, 29]. Previous research indicates that the care needs of older adults have a substantial negative impact on household finances, which in turn limits the use of medical services [49, 50]. UMNs are becoming an increasingly important issue, especially for poorer older individuals whose families often lack the resources and support to meet their medical needs in the absence of a comprehensive government safety net [3]. In the preliminary model of our study, older individuals with higher education levels and higher household per capita income tended to report fewer UMNs. However, after adjusting for other potential factors and health-related variables, the bivariate associations weakened, especially the impact of education level. This finding suggests that socioeconomic advantages may mitigate the impact of UMNs among the older adults, or conversely, the impact of socioeconomic disadvantages on unmet medical needs may be exacerbated by health issues. This aligns with our understanding of the relationship between socioeconomic disadvantage and health [51, 52], as well as the cumulative effects of economic hardship over the life course. Regarding the equity of UMNs, the findings reveal a pro-poor inequality, as previously reported, with older adults with lower socioeconomic status are more likely to experience UMNs [3, 53, 54]. These findings highlight the persistent role of economic factors in shaping UMNs.

Additionally, we observed that the presence or absence of medical insurance did not show significant differences in the impact on UMNs. A possible explanation for this is that China has essentially achieved universal health insurance coverage, with basic medical insurance covering over 95% of the population [4]. This distribution in the sample may result in a lack of significant findings in the analysis. However, it is important to note that the current universal health insurance in China aims to provide “basic coverage”, meaning the level of coverage remains relatively low. Given the current depth and breadth of insurance coverage (in terms of reimbursement scope and rates), it is difficult to fully address the complex medical challenges faced by the older adults. Socioeconomically disadvantaged groups may forgo treatment due to limited affordability. In other words, medical insurance alone cannot entirely compensate for individuals’ socioeconomic limitations when medical services are needed. This is also reflected in the results of the disparity analysis—among older individuals reporting UMNs, economic hardship was the primary reason regardless of their insurance status. According to the 2023 WHO Global Monitoring Report, while China has made remarkable achievements in improving the health of its residents, the rate of catastrophic health expenditure remains higher than the global average (13.5%) and has been worsening in recent years [55]. This indicates that China’s universal health insurance coverage network still has a long way to go.

Third, the need-related factors associated with UMNs and inequalities are the number of chronic diseases and independence in activities of daily living. Our findings are consistent with previous research indicating that individuals with multiple chronic conditions and functional limitations are more likely to report UMNs [35]. Our study also reveals that for each additional chronic disease, the likelihood of older adults experiencing UMNs increases exponentially. This may be attributed to the fact that individuals with chronic diseases often require long-term or lifelong treatment and medication, which can lead to a greater likelihood of UMNs compared to those without chronic conditions. As in other countries, China’s current healthcare system still faces the significant challenge of providing age-appropriate, long-term, and comprehensive care for individuals with multimorbidity [30,56].

Overall, this study estimates UMNs among the older adults and identifies significant gaps between demand and provision, particularly concentrated in key groups. Given the accelerated aging process in China, policy changes targeting underserved populations are urgently needed. Addressing UMNs requires ensuring that individuals with medical needs have access to appropriate medical services. First, from the perspective of regional equity, policies should focus more on reforming the current healthcare system to redistribute medical resources to underdeveloped and rural areas, improving the accessibility of medical services to meet health needs and narrowing the regional health gap. However, such redistribution faces real implementation challenges: qualified health workers tend to cluster in urban tertiary hospitals, less-developed regions often lack the fiscal capacity to sustain new services after central transfers taper off, and well-resourced urban institutions may resist reallocation. Feasible policy levers include differentiated salary and housing subsidies for medical staff willing to practice in western and central Hainan, strengthening tiered diagnosis and treatment and medical-consortium arrangements to share specialists and referral pathways between eastern tertiary hospitals and underserved primary facilities, expanding telemedicine and digital health tools (such as remote monitoring and mobile health applications) as a partial substitute for relocating specialist services, and embedding equity-oriented indicators into the performance evaluation of local health authorities.

Second, from the perspective of system equity, increased investment in the healthcare system is needed to better meet the health needs of the older adults, particularly those who are socioeconomically vulnerable and those with multiple health issues. For socioeconomically vulnerable older adults, additional investment should be directed toward strengthening the benefit package of basic medical insurance by raising reimbursement ceilings, lowering deductibles, and expanding coverage for outpatient services and chronic disease medications, together with targeted medical financial assistance for low-income households. Our finding that economic hardship remained the primary reason for unmet medical needs regardless of insurance status indicates that expanding coverage without sufficient depth is inadequate. For older adults with multiple chronic conditions, investment is most effective when focused on integrated and continuous care, including primary care and family doctor (general practitioner) based chronic disease management, team-based coordination across specialties, and shared electronic records, rather than on expanding episodic specialist services, as the current health care delivery system and social security system has limitations in addressing the burdens and complex needs associated with multimorbidity [3].

Third, from the perspective of improving healthcare experiences or enhancing service quality. Dissatisfaction with care experiences may discourage individuals from seeking further treatment [57]. Additionally, UMNs may persist despite high rates of service utilization if these services fall short in any aspect of quality, effectiveness, relevance, or appropriateness [6]. Professional self-regulation is a policy tool that is seldom emphasized. The current healthcare system is a social system that still needs to work towards improving clinical standards and promoting patient quality and safety [13]. Fourth, from the perspective of patient empowerment. There is a need to enhance the capabilities of patients beyond government and healthcare professionals. Studies show that the health literacy and awareness of older residents need to be improved, necessitating interventions to promote health education and awareness. Empowering patients to make more informed choices will have a positive impact on health outcomes and patient satisfaction [13].

This study has several limitations. First, the results may not be easily generalized, as they could be influenced by cultural background, social, and healthcare system contexts. Therefore, it is necessary to interpret the research findings in light of the characteristics of the research setting in future studies. Additionally, the data used are based on participants’ self-reports, and the accuracy of the survey data may be affected by any number of bias sources. Finally, due to the cross-sectional study design used in this research, causal relationships cannot be established, future research may utilize cumulative longitudinal data to confirm causal relationships. Nevertheless, we have provided a preliminary evidence base for further research.

## Conclusion

This study indicates that a considerable proportion of the older adults in China have unmet medical needs. Our findings support the view that efforts to achieve equity in the provision of medical services should continue and remain a significant policy concern, especially in the context of the strategic pursuit of healthy aging and universal health coverage. Specifically, policy interventions should focus on expanding the coverage of medical services and address financial and non-financial barriers to accessing medical services, especially for poor and vulnerable individuals living in areas with fewer medical resources. Future research is needed to address the challenges of monitoring the evolution of UMNs and identifying their determinants and inequalities.

## Data Availability

The datasets used in the current study are not publicly available due to the confidentiality policy but are available from the corresponding author on reasonable request.

## Abbreviations

UHC: Universal health coverage
UMNs: Unmet medical needs
NHSS: National Health Service Survey
ADL: Activities of daily living
CI: Concentration index
